# pH-Regulated Cation-Dependent Electrochromism of Electrodeposited WO_3_·2H_2_O Films in Aqueous Electrolytes

**DOI:** 10.3390/ma19101943

**Published:** 2026-05-09

**Authors:** Ruoming Du, Aihua Yao

**Affiliations:** School of Materials Science and Engineering, Tongji University, Shanghai 201804, China; 2331530@tongji.edu.cn

**Keywords:** electrochromism, hydrated WO_3_ films, aqueous electrolyte, pH regulation

## Abstract

**Highlights:**

**What are the main findings?**

- WO_3_·2H_2_O films exhibit pronounced pH-dependent electrochromism in aqueous electrolytes.
- Lower-pH LiCl provides the best overall electrochromic performance in the Li^+^ system.
- ZnCl_2_ shows stronger pH-dependent kinetic limitations than LiCl.

**What are the implications of the main findings?**

- The pH effect in hydrated WO_3_ depends strongly on the cation involved.
- Multivalent-ion systems are more sensitive to electrolyte-controlled transport and interfacial conditions.
- pH control offers a practical route to improving aqueous WO_3_-based electrochromic systems.

**Abstract:**

Aqueous electrochromic systems based on tungsten oxide (WO_3_) have attracted increasing attention because of their high ionic conductivity, low cost, and improved safety compared with organic systems. However, the role of electrolyte pH in regulating the electrochromic behavior of hydrated WO_3_ films remains insufficiently understood, particularly across cation systems with different valences. In this work, amorphous WO_3_·2H_2_O films were electrodeposited on ITO substrates and systematically evaluated in LiCl and ZnCl_2_ aqueous electrolytes with different pH values, with acidic AlCl_3_ used as a supplementary trivalent system. The results reveal pronounced pH-dependent electrochromic behavior in both the monovalent and divalent systems. In LiCl, acidic conditions, especially pH 2.0, gave the best overall performance, including high optical modulation and improved cycling stability, while the dominant pseudocapacitive charge-storage behavior was largely preserved. In ZnCl_2_, films tested at pH 1.5–2.0 showed significantly better electrochromic performance than those at higher pH values, indicating a much stronger kinetic sensitivity to pH. Combined experimental and first-principles results show that electrolyte pH influences not only proton availability, but also the cation-dependent interfacial charge-compensation environment in hydrated WO_3_ films.

## 1. Introduction

Electrochromic materials can reversibly modulate their optical properties under an applied electric field and have attracted extensive attention for applications in smart windows, displays, anti-glare mirrors, and energy-saving devices [1]. Among various electrochromic materials, tungsten oxide (WO_3_) is one of the most widely studied because of its large optical contrast, good reversibility, and tunable coloration behavior [2]. In particular, hydrated WO_3_ has received increasing interest in recent years. Owing to their open structure and the presence of structural water, hydrated WO_3_ can provide more accessible ion-transport pathways, facilitate proton-coupled charge compensation, and better accommodate reversible structural changes during electrochemical switching [3,4,5]. These characteristics make hydrated WO_3_ especially promising for aqueous electrochromic systems.

Compared with organic electrolytes, aqueous electrolytes offer several practical advantages, including high ionic conductivity, low cost, and improved safety [6]. More importantly, aqueous media provide broad opportunities to regulate electrochromic performance through electrolyte composition, cation species, concentration, and pH [6,7,8,9]. Recent studies have shown that hydrated or amorphous WO_3_ films can exhibit fast switching, large optical modulation, and good cycling stability in aqueous systems, highlighting their potential for practical electrochromic applications [6,8,9].

Despite this progress, the electrochromic behavior of hydrated WO_3_ in aqueous electrolytes is still not sufficiently understood from the perspective of the coupled effects of electrolyte pH and cation chemistry. Most previous studies have focused on a single electrolyte or a single cation system, while systematic investigations covering both electrolyte pH and cation valence remain limited. As a result, the extent to which the pH-regulated electrochromic response of hydrated WO_3_ is governed by the identity and valence of the charge-compensating cation remains unclear. This issue is scientifically important because pH can influence not only proton availability, but also the interfacial electrochemical environment, electric-double-layer structure, and the transfer barrier for solvated ions [10,11]. At the same time, monovalent, divalent, and trivalent cations differ substantially in charge density, hydration structure, and interaction with the W-O host framework, which may lead to distinct insertion kinetics, charge-storage characteristics, and optical modulation behaviors [11,12,13].

For hydrated WO_3_, these effects are expected to be particularly significant because structural water can participate in proton transport and modify the local environment for the insertion of other cations [3,4,5]. Therefore, the electrochromic response in aqueous electrolytes is unlikely to be determined by a single factor such as proton insertion or cation valence alone; instead, it is expected to arise from the coupled effects of proton activity, cation hydration/desolvation behavior, interfacial ion transport, and the structural adaptability of the host framework. A systematic investigation of aqueous electrolytes containing cations with different valences is therefore essential to clarify how these factors collectively regulate the electrochromic behavior of hydrated WO_3_.

In this work, amorphous WO_3_·2H_2_O films electrodeposited on ITO substrates were used as a model system to investigate pH-regulated cation-dependent electrochromism in aqueous electrolytes. LiCl and ZnCl_2_ were selected as representative monovalent and divalent electrolytes, respectively, and their pH values were systematically varied to examine the effects of pH on electrochemical charge storage, optical modulation, switching kinetics, and cycling stability. In addition, an acidic AlCl_3_ electrolyte was introduced as a supplementary trivalent system for comparison. By combining electrochemical and electrochromic characterization with first-principles calculations, we show that electrolyte pH governs not only proton participation, but also the cation-dependent interfacial charge-compensation environment in WO_3_·2H_2_O. The results further reveal that the influence of pH is relatively moderate in the Li^+^ system, where the dominant pseudocapacitive behavior is preserved, but becomes more pronounced in the Zn^2+^ system, where pH strongly affects insertion kinetics and electrochromic accessibility. These findings provide new insight into the coupled roles of pH and cation chemistry in aqueous WO_3_ electrochromism.

## 2. Materials and Methods

### 2.1. Materials

Tungsten powder (99.98%), hydrogen peroxide solution (30 wt.%), lithium chloride (LiCl), aluminum chloride (AlCl_3_), zinc chloride (ZnCl_2_), hydrochloric acid (HCl), sodium hydroxide (NaOH), acetone, absolute ethanol, and isopropanol were all purchased from Aladdin Reagent Co., Ltd. (Shanghai, China), and used as received without further purification. Indium tin oxide (ITO)-coated glass substrates (2 cm × 5 cm, sheet resistance: 7–10 Ω/sq) were purchased from Suzhou Shangyang Solar Technology Co., Ltd. (Suzhou, China), and used as the conductive substrates for film deposition.

### 2.2. Film Preparation and Electrolytes

Prior to deposition, the ITO-coated glass substrates were cleaned sequentially in ethanol, acetone, and deionized water, followed by drying in air. Amorphous WO_3_·2H_2_O films were then electrodeposited on the cleaned ITO substrates by a potentiostatic method using the precursor solution reported in our previous work [14]. Briefly, tungsten powder was dissolved in H_2_O_2_ to form a peroxotungstic acid-based deposition solution, which was then aged for 36 h at 25 °C before use. Electrodeposition was carried out in a conventional three-electrode system using the ITO substrate as the working electrode, a platinum sheet as the counter electrode, and an Ag/AgCl electrode as the reference electrode. The deposition was performed at a constant potential of −0.5 V for 30 min. After deposition, the films were rinsed with deionized water and dried at 25 °C before further characterization and electrochemical measurements. Under the same deposition conditions, the film thickness was approximately 200 nm in our previous work [14]. Detailed information on the precursor preparation and film deposition process is provided in Ref. [14].

For the Li^+^ system, 1.0 mol/L LiCl aqueous solutions with pH values of 2.0, 5.0, and 8.6 were used. For the Zn^2+^ system, 0.5 mol/L ZnCl_2_ aqueous solutions with pH values of 1.5, 2.0, 2.5, and 3.0 were prepared. For comparison, 0.33 mol/L AlCl_3_ aqueous electrolyte with a measured pH of about 2.3 was also used as a supplementary trivalent system. The electrolyte concentrations were selected to maintain the same total cation charge concentration among the different systems. For the LiCl system, the electrolyte pH was adjusted using dilute HCl or NaOH, whereas for the ZnCl_2_ system, the pH was adjusted using dilute HCl. Because only a small amount of dilute NaOH was used for pH adjustment, the influence of the introduced Na^+^ on the electrochemical and electrochromic comparison is expected to be minimal. All pH values were measured using a calibrated pH meter (PHS-3C, LEICI, INESA Scientific Instrument Co., Ltd., Shanghai, China) before testing. All aqueous solutions were prepared with deionized water.

### 2.3. Characterization

The surface morphology of the films was examined using a field-emission scanning electron microscope (FESEM, S-4800, Hitachi, Tokyo, Japan). Raman spectra were collected using a laser confocal Raman microscope (LabRAM HR Evolution, HORIBA Scientific, HORIBA Ltd., Kyoto, Japan) with a 514 nm excitation laser to analyze the structural features of the films. X-ray photoelectron spectroscopy (XPS) measurements were performed using an X-ray photoelectron spectrometer (ESCALAB 250Xi, Thermo Fisher Scientific, Shanghai, China) to analyze the chemical states of W in selected cycled samples. The concentration of dissolved W in the electrolyte after cycling was analyzed by inductively coupled plasma optical emission spectroscopy (ICP-OES, Optima 8300, PerkinElmer, Waltham, MA, USA).To further verify the structural characteristics of the films used in the present study, representative XRD patterns of the bare ITO substrate and the as-deposited film on ITO were provided in the Supplementary Materials (Figure S1). The structural assignment of the electrodeposited film as amorphous WO_3_·2H_2_O was based on the same preparation protocol established in our previous work [14], where XRD, XPS, Raman, SEM, and thickness analyses were systematically performed.

### 2.4. Electrochemical and Electrochromic Measurements

Electrochemical and electrochromic measurements were carried out in a conventional three-electrode system, with the film-coated ITO substrate as the working electrode, a platinum sheet as the counter electrode, and an Ag/AgCl electrode as the reference electrode. Unless otherwise stated, all potentials reported in this work are referenced to Ag/AgCl (saturated KCl).

Electrochemical characterization was performed using an electrochemical workstation (CHI660D, Shanghai Chenhua Instrument Co., Ltd., Shanghai, China) by cyclic voltammetry (CV), chronoamperometry (CA) and chronocoulometry (CC). CV measurements were conducted over the potential range from −0.5 to +0.3 V at various scan rates. CA measurements were performed by applying alternating potential steps of −0.5 V for 20 s and +0.3 V for 20 s to determine the switching response times. CC measurements were carried out under the same potential-step conditions, with each potential held for 20 s. Optical transmittance was measured using a fiber-optic spectrometer (S2000-VIS, Ocean Optics, Dunedin, FL, USA) over the wavelength range of 400–1100 nm and the transmittance at 633 nm was used for quantitative comparison of electrochromic performance. The switching response time was defined as the time required to reach 90% of the full transmittance modulation. Cycling stability was evaluated by repeated double-step CA measurements, in which the potential was alternated between −0.5 V and +0.3 V with a holding time of 20 s at each potential. The transmittance changes were monitored in situ during electrochemical switching. Open-circuit optical memory measurements were performed in aqueous 1.0 mol/L LiCl electrolytes at pH 2.0 and 8.6, as well as in 0.5 mol/L ZnCl_2_ electrolytes at pH 2.0 and 3.0. Prior to the open-circuit test, the WO_3_·2H_2_O film was colored by applying a potential of −0.5 V for 20 s. The external bias was then removed, and subsequently transmittance at 633 nm was monitored in situ under open-circuit conditions to assess the retention of the colored state.

The optical modulation (Δ*T*), bleaching/coloration times (*t*_b_/*t*_c_), coloration efficiency (CE), and cycling stability were used to evaluate the electrochromic performance. The coloration efficiency was calculated according to:

$$CE=\frac{∆OD}{∆Q}$$

where ΔOD is the change in optical density and Δ*Q* is the inserted charge density during coloration. The optical density change was determined from

$$∆OD=log\left(T_{b}/T_{c}\right)$$

where *T*_b_ and *T*_c_ are the transmittances of the bleached and colored states, respectively.

To further evaluate the pseudocapacitive charge-storage capability of the WO_3_·2H_2_O films, the areal capacitance was calculated from the coloration charge density obtained during the potential-step CC measurements. The net coloration charge density was obtained by subtracting the initial charge density from the maximum charge density during the coloration step. The areal capacitance *C_A_* was then calculated according to

$$C_{A}=\frac{Q}{∆V}$$

where *Q* is the net coloration charge density and ∆*V* is the applied potential window. Since the potential was switched between −0.5 and +0.3 V vs. Ag/AgCl, ∆V was 0.8 V.

### 2.5. Kinetic Analysis

The relationship between the peak current and scan rate was analyzed using the power-law equation [15]:

$$i=av^{b}$$

where *a* and *b* are adjustable parameters. A *b* value close to 0.5 indicates a diffusion-controlled process, whereas a value close to 1.0 suggests a surface-controlled capacitive process. The capacitive and diffusion-controlled contributions were further analyzed according to the following equation [15]:

$$i\left(V\right)=k_{1}v+k_{2}v^{1/2}$$

where *k*_1_*v* and *k*_2_*v*^1/2^ represent the capacitive and diffusion-controlled contributions, respectively. For the Zn^2+^ electrolyte system, the apparent diffusion coefficient was further estimated using the Randles-Sevcik relationship [16].

### 2.6. First-Principles Calculations

First-principles calculations were performed to evaluate the intrinsic insertion energetics of different cations in WO_3_·2H_2_O. The initial WO_3_·2H_2_O structural model was constructed from a previously reported isostructural MoO_3_·2H_2_O framework and then fully optimized [17]. A fully relaxed (WO_3_·2H_2_O)_8_ supercell was used as the host model, and a single Li, Zn, or Al ion was placed at several candidate sites within the intralayer framework and hydrated channels. Each initial configuration was then fully optimized to identify locally stable insertion sites. The insertion energy was calculated as:

$$∆E=E\left[M{\left(WO_{3}\cdot 2H_{2}O\right)}_{8}\right]-E\left[{\left(WO_{3}\cdot 2H_{2}O\right)}_{8}\right]-\mu_{M}$$

where E[M(WO_3_∙2H_2_O)_8_] and E[(WO_3_∙2H_2_O)_8_ ] are the total energies of the ion-inserted and pristine supercells, respectively, and *μ*_M_ is the chemical potential of the inserted species (M = Li, Zn, Al) referenced to the corresponding elemental bulk phase. Density functional theory (DFT) calculations were carried out using the Vienna Ab initio Simulation Package (VASP, version 6.4.2). The exchange-correlation energy was described by the generalized gradient approximation with the Perdew-Burke-Ernzerhof (PBE) functional [18], and the ion-electron interaction was treated using the projector augmented-wave (PAW) method [19]. Further computational details are provided in the Supplementary Materials.

## 3. Results

### 3.1. LiCl Electrolyte

Because amorphous WO_3_·2H_2_O possesses a hydrated framework favorable for ion transport, its electrochromic behavior is expected to be sensitive to electrolyte pH, which can influence proton availability and the interfacial charge-compensation environment. To examine this effect, the electrochemical and electrochromic properties of the film were investigated in 1.0 mol/L LiCl aqueous electrolytes with pH values of 2.0, 5.0, and 8.6.

**Figure 1:**
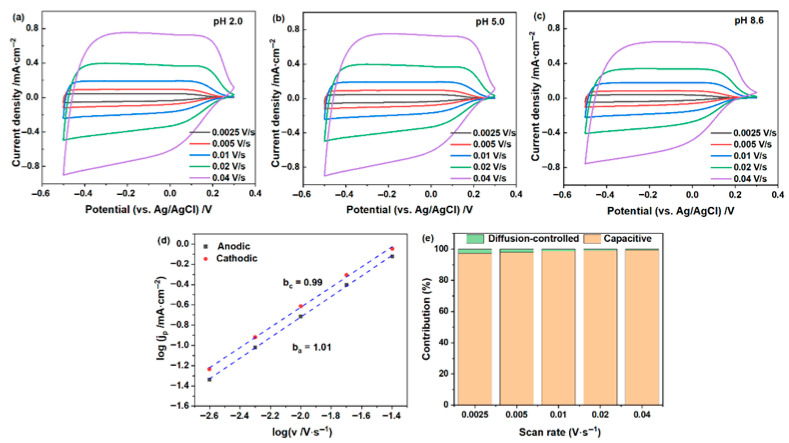
Electrochemical behavior of the WO_3_·2H_2_O film in 1.0 mol/L LiCl electrolytes with different pH values. (**a**–**c**) CV curves recorded at different scan rates at pH 2.0, 5.0, and 8.6, respectively; (**d**) log(i)-log(v) fitting of anodic and cathodic peak currents at pH 2.0; (**e**) capacitive and diffusion-controlled contributions at different scan rates for the film tested at pH 2.0.

As shown in Figure 1a–c, the cyclic voltammograms (CVs) of the WO_3_·2H_2_O film recorded at different scan rates exhibit broad quasi-rectangular profiles under all investigated pH conditions, indicating predominantly pseudocapacitive behavior. The film tested at pH 2.0 delivers the highest current density and largest integrated CV area, while the electrochemical response decreases progressively with increasing pH. Kinetic analysis in Figure 1d,e for the representative pH 2.0 sample shows that both the anodic and cathodic b values are close to 1.0, and the capacitive contribution is dominant. Similar trends were also observed at pH 5.0 and 8.6. These results suggest that, in the LiCl electrolyte, varying pH does not fundamentally alter the dominant charge-storage mechanism of the hydrated WO_3_ film. Instead, pH mainly regulates the accessible extent of electrochemical charging, while the predominant pseudocapacitive character is preserved.

**Figure 2:**
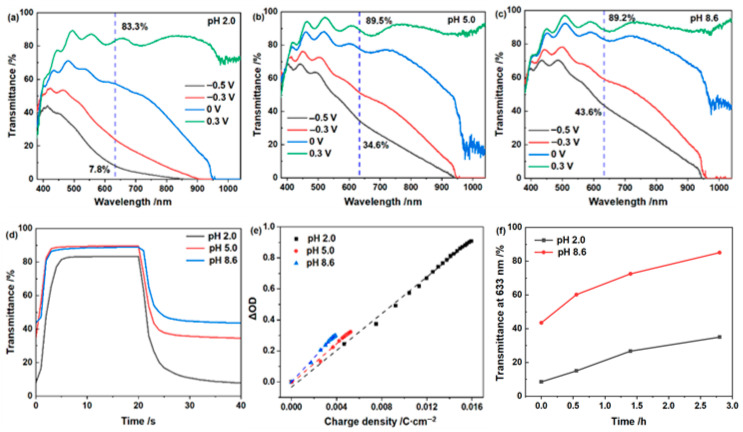
Electrochromic performance of the WO_3_·2H_2_O film in 1.0 mol/L LiCl electrolytes with different pH values: (**a**–**c**) in situ transmittance spectra recorded under different applied potentials at pH 2.0, 5.0, and 8.6, respectively; (**d**) real-time transmittance response at 633 nm; (**e**) coloration efficiency determined from the relationship between optical density change and charge density; (**f**) open-circuit optical memory behavior at 633 nm after coloration at −0.5 V.

The electrochromic response, however, shows a strong dependence on electrolyte pH. As summarized in Table 1 and Figure 2, the WO_3_·2H_2_O film exhibits the largest optical modulation (∆*T*) at 633 nm in the pH 2.0 electrolyte, reaching 75.5%, and the modulation gradually decreases with increasing pH. Consistently, the areal capacitance calculated from the coloration charge density decreases from 26.1 mF/cm^2^ at pH 2.0 to 12.6 and 9.70 mF/cm^2^ at pH 5.0 and 8.6, respectively, indicating that acidic LiCl electrolyte increases the accessible pseudocapacitive charge-storage capacity of the film. In contrast, the coloration efficiency (CE) increases from 59.2 to 78.1 cm^2^/C as the pH increases from 2.0 to 8.6, showing that deeper coloration and larger charge storage do not necessarily correspond to more efficient charge utilization. From an electronic-level perspective, WO_3_ coloration originates from electron injection into W 5d-derived states and the partial reduction in W^6+^ to W^5+^, forming localized polaronic states responsible for optical absorption. At low pH, more W^5+^-related colored sites are generated, giving a large Δ*T*. However, because effective optical absorption is generally associated with intervalence or polaronic transitions between neighboring W^5+^ and W^6+^ sites [20], a high density of W^5+^ sites may make the optical absorption no longer increase proportionally with the inserted charge, leading to a lower CE despite the larger charge-storage capacity. In addition, proton-insertion-coupled electron transfer in WO_3_ under acidic conditions may be accompanied by hydrogen evolution, indicating that part of the injected charge could be consumed by H^+^ reduction rather than effective coloration [21].

The switching data further support this interpretation. Although the film in the pH 2.0 electrolyte exhibits the largest ∆*T* and areal capacitance, it does not exhibit the fastest switching response. This result suggests that the electrolyte condition favorable for deep coloration and large pseudocapacitive charge storage is not necessarily optimal for switching kinetics or charge-utilization efficiency. Therefore, pH optimization in the LiCl system requires balancing optical contrast, charge efficiency, and switching kinetics. The open-circuit stability also depends strongly on electrolyte pH. As shown in Figure 2f, after removing the applied bias, the film tested at pH 2.0 remains colored for a longer time, while the film tested at pH 8.6 quickly returns to a more transparent state. This comparison demonstrates that the acidic LiCl electrolyte provides better retention of the colored state under open-circuit conditions.

**Figure 3:**
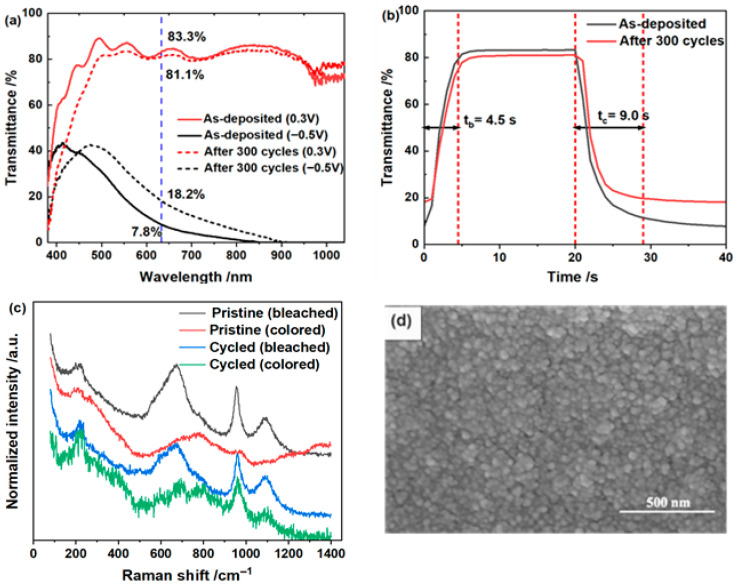
Cycling stability and post-cycling characterization of the WO_3_·2H_2_O film in 1.0 mol/L LiCl electrolyte at pH 2.0. (**a**) In situ transmittance spectra of the as-deposited film and the film after 300 cycles under bleached (+0.3 V) and colored (−0.5 V) states; (**b**) real-time transmittance response at 633 nm before and after 300 cycles; (**c**) normalized Raman spectra of the pristine and cycled films in the bleached and colored states; (**d**) SEM image of the film after 300 cycles.

The cycling results further show that acidic conditions are more favorable for durability in the LiCl system. As listed in Table 1 and shown in Figure 3, the film tested at pH 2.0 maintains substantial ∆*T* over hundreds of cycles, whereas the higher-pH samples degrade much more rapidly within tens of cycles. The Raman spectra in Figure 3c show that the main W-O-related vibrational features remain observable after cycling, although the bands become weaker and broader, especially in the colored state. This result indicates that the WO_3_·2H_2_O framework is generally retained after repeated switching, accompanied by some local structural disorder or surface reconstruction. Consistently, the SEM image in Figure 3d shows that the cycled film maintains a relatively uniform and compact morphology. In addition, ICP-OES analysis of the electrolyte after cycling detected W only at the ppm level, suggesting that dissolution of the WO_3_·2H_2_O film was limited within the present cycling period. These observations indicate that the hydrated WO_3_ film retains better electrochromic reversibility and structural stability under acidic LiCl conditions. Additional post-cycling characterization of the higher-pH samples is provided in Figure S2. Overall, the LiCl results show that lowering the electrolyte pH enhances the accessible electrochromic response and improves cycling stability, while preserving the predominantly pseudocapacitive nature of charge storage.

### 3.2. ZnCl_2_ Electrolyte

To determine whether the pH-dependent behavior observed in the LiCl electrolyte is extended to multivalent-ion systems, WO_3_·2H_2_O films were further evaluated in 0.5 mol/L ZnCl_2_ aqueous electrolytes with pH values of 1.5, 2.0, 2.5, and 3.0. The ZnCl_2_ system was examined within an acidic pH window because higher-pH Zn^2+^ electrolytes are less suitable for controlled comparison due to possible hydrolysis-induced changes in electrolyte speciation.

**Figure 4:**
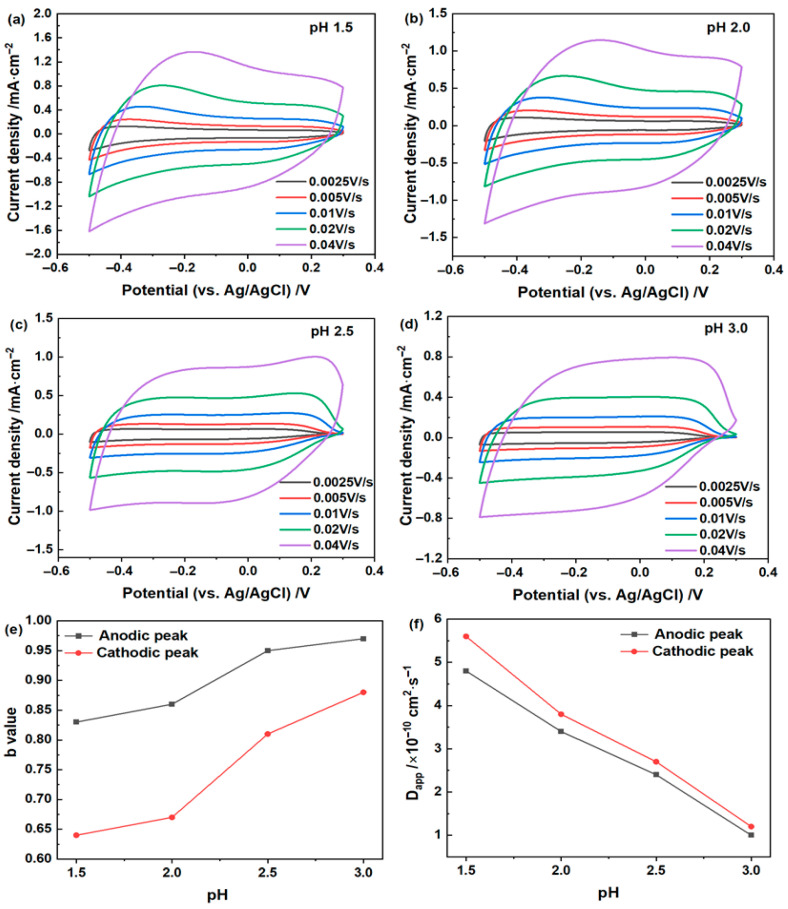
Electrochemical and kinetic analysis of the WO_3_·2H_2_O film in 0.5 mol/L ZnCl_2_ electrolytes with different pH values. (**a**–**d**) CV curves recorded at different scan rates at pH 1.5, 2.0, 2.5, and 3.0, respectively. (**e**) Variation in the anodic and cathodic b values with pH. (**f**) Apparent diffusion coefficients (*D*_app_) derived from the anodic and cathodic peaks as a function of pH.

As in the LiCl system, the CV curves in Figure 4a–d exhibit broad pseudocapacitive features under all investigated pH conditions. However, the kinetic response of the ZnCl_2_ system shows a much stronger dependence on pH. Both the current response and the integrated CV area decrease progressively with increasing pH, with the largest CV area obtained at pH 1.5. More importantly, unlike in the LiCl electrolyte, the calculated b values vary markedly with pH. As shown in Figure 4e, under lower-pH conditions the b values fall between 0.5 and 1.0, indicating a mixed diffusion-controlled and capacitive process. With increasing pH, the b values approach 1.0, suggesting that the electrochemical response becomes increasingly dominated by surface or near-surface processes. Consistent with this trend, the calculated apparent diffusion coefficient (*D*_app_) decreases continuously from pH 1.5 to 3.0 (Figure 4f). These results indicate that the ZnCl_2_ system is much more kinetically sensitive to pH than the LiCl system. A similar pH dependence has also been reported for amorphous hydrated WO_3_ in Zn-based aqueous electrolytes, where lowering the electrolyte pH improved electrochemical accessibility and cycling stability [22].

**Figure 5:**
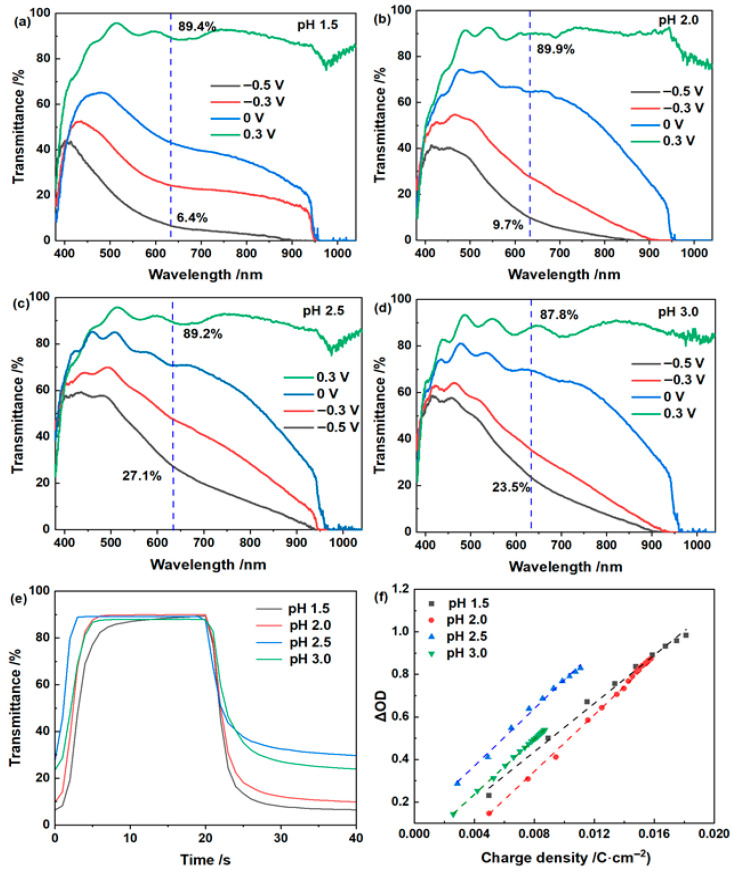
Electrochromic performance of the WO_3_·2H_2_O film in 0.5 mol/L ZnCl_2_ electrolytes with different pH values: (**a**–**d**) in situ transmittance spectra recorded under different applied potentials at pH 1.5, 2.0, 2.5 and 3.0, respectively; (**e**) real-time transmittance response at 633 nm; (**f**) coloration efficiency determined from the relationship between optical density change and charge density.

The electrochromic performance of the WO_3_·2H_2_O films in the ZnCl_2_ electrolyte also shows a pronounced pH dependence (Table 2 and Figure 5). The ∆*T* at 633 nm reaches 83.0% at pH 1.5 and 80.2% at pH 2.0, both markedly higher than those at pH 2.5 and 3.0. A similar trend is observed for the areal capacitance, which decreases from 32.9 mF/cm^2^ at pH 1.5 to 16.6 mF/cm^2^ at pH 3.0. This indicates that lower-pH ZnCl_2_ electrolytes promote not only deeper electrochromic modulation but also more accessible pseudocapacitive charge storage. In terms of switching kinetics, however, the sample at pH 2.0 appears to provide the best overall balance, whereas the film at pH 1.5, despite its slightly larger modulation amplitude and higher areal capacitance, exhibits slower bleaching. The CE varies less significantly than the ∆*T*, with the highest value observed at pH 2.5. Open-circuit optical memory measurements for representative ZnCl_2_ electrolytes are shown in Figure S3. After removal of the applied bias, the pH 2.0 sample shows better retention of the colored state than the pH 3.0 sample. This result further confirms the beneficial role of lower-pH ZnCl_2_ electrolyte in improving open-circuit stability.

As in the LiCl system, these results indicate that different electrochromic metrics do not necessarily reach their optimum values under the same electrolyte condition. More importantly, the marked decrease in Δ*T* at higher pH, together with the changes in the *b* values and *D*_app_, suggests that increasing pH not only weakens the electrochromic response and charge-storage capacity, but also makes it harder for Zn^2+^-related charge compensation to proceed throughout the bulk of the hydrated WO_3_ film. As a result, the reaction becomes increasingly confined to the surface or near-surface region.

**Figure 6:**
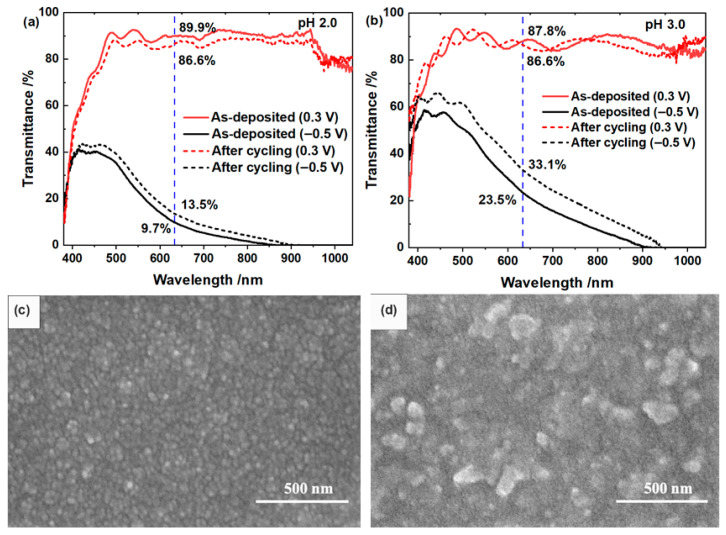
Post-cycling electrochromic and morphological comparison of WO_3_·2H_2_O films in 0.5 mol/L ZnCl_2_ electrolytes at different pH values. (**a**) In situ transmittance spectra of the as-deposited film and the film after 400 cycles at pH 2.0 in the bleached (+0.3 V) and colored (−0.5 V) states. (**b**) In situ transmittance spectra of the as-deposited film and the film after 150 cycles at pH 3.0 in the bleached (+0.3 V) and colored (−0.5 V) states. (**c**) SEM image of the cycled film at pH 2.0. (**d**) SEM image of the cycled film at pH 3.0.

Cycling tests further show that lower-pH conditions are more favorable for durability in the ZnCl_2_ electrolyte. As summarized in Table 2, the films tested at pH 1.5 and 2.0 retain about 91% of their initial ∆*T* after 400 cycles, whereas the samples at pH 2.5 and 3.0 show much faster attenuation. The post-cycling transmittance spectra in Figure 6a,b further illustrate this difference: the film tested at pH 2.0 still maintains a relatively large ∆*T* after 400 cycles, whereas the film tested at pH 3.0 shows more pronounced fading after only 150 cycles. Consistently, the SEM images in Figure 6c,d show that the cycled film at pH 2.0 retains a more uniform and compact morphology, while the film tested at pH 3.0 exhibits obvious aggregation and surface coarsening. Additional post-cycling characterization for the pH 1.5 and 2.5 samples is provided in Figure S4. Notably, although dissolution of amorphous WO_3_ in strongly acidic aqueous media can be a concern, the film tested at the most acidic condition of pH 1.5 still retained 90.9% of its initial optical modulation after 400 cycles. The corresponding post-cycling SEM image also shows a relatively uniform surface morphology without obvious film dissolution, cracking, or delamination. Additional Raman and W 4f XPS characterization of the film after 400 cycles at pH 1.5 further confirms that the W-O-related vibrational features and dominant W^6+^ signals are retained after cycling. These results indicate that lower-pH ZnCl_2_ electrolytes are effective in maintaining reversible electrochromic activity and structural integrity during repeated cycling.

### 3.3. Comparison of Different Cation Systems and Mechanistic Implications

The results obtained in the LiCl and ZnCl_2_ electrolytes reveal a clear cation-dependent difference in pH sensitivity. Lower-pH conditions improve the electrochromic performance in both systems, but the effect is more pronounced in the ZnCl_2_ electrolyte. In the LiCl system, pH mainly regulates the accessible extent of electrochemical charging while preserving the dominant pseudocapacitive character. In contrast, in the ZnCl_2_ electrolyte, pH also affects how easily ions can participate in electrochromic insertion within the bulk of the film, with the response evolving from a mixed diffusion-controlled/capacitive process at lower pH to a more surface- or near-surface-dominated process at higher pH. It should be noted that the investigated pH windows of the LiCl and ZnCl_2_ electrolytes are different because of their different electrolyte chemistry and hydrolysis behavior. Nevertheless, both systems include a common acidic condition at pH 2.0, which provides a direct reference point for comparing the Li^+^ and Zn^2+^ responses under the same pH condition. These observations indicate that the influence of electrolyte pH on hydrated WO_3_ is intrinsically cation-dependent.

The pH-dependent response may first be related to changes in the surface protonation state and electric-double-layer structure at the hydrated WO_3_/electrolyte interface. Hydrated WO_3_ surfaces contain amphoteric W-OH groups, whose protonation/deprotonation state depends on the electrolyte pH relative to the point of zero charge (PZC). Although the PZC of the present WO_3_·2H_2_O films was not directly measured, lower pH is expected to create a more proton-rich interfacial environment, which favors proton-assisted charge compensation. In contrast, higher pH may increase the fraction of deprotonated surface sites and alter cation accumulation, desolvation, and interfacial transfer. This interfacial effect is schematically illustrated in Figure S6.

Beyond the interfacial protonation effect, the cation-dependent pH sensitivity can be further understood in terms of the coupled effects of proton participation, structural water, and cation-dependent ion transport in WO_3_·2H_2_O. The hydrated WO_3_ framework contains structural water, which may facilitate proton transfer through local hydrogen-bonding networks [3,5], allowing possible Grotthuss-like proton hopping under low-pH conditions where proton availability is high. This is consistent with the larger CV area, stronger optical modulation, and better cycling stability observed under acidic conditions. Previous operando XRD and spectroscopic studies have shown that confined structural water in tungsten oxide hydrates promotes reversible proton intercalation and improves structural stability during electrochemical cycling [5,20], supporting the role of structural water in proton-assisted charge compensation and framework adaptability.

In the ZnCl_2_ electrolyte, possible H^+^/Zn^2+^ co-intercalation or proton-assisted Zn^2+^ insertion should also be considered. At lower pH, abundant protons may participate in charge compensation and partially screen the strong electrostatic interaction associated with divalent Zn^2+^, thereby facilitating interfacial transfer and bulk electrochromic accessibility. In contrast, increasing pH reduces proton availability and makes Zn^2+^-related charge compensation more dependent on the desolvation and transport of hydrated Zn^2+^ species. Compared with Li^+^, Zn^2+^ has stronger hydration and a higher interfacial transfer barrier, making its electrochromic response more sensitive to pH and the local electrolyte environment. Therefore, the electrolyte effect in multivalent-ion systems should be viewed as the combined result of proton participation, possible H^+^/Zn^2+^ co-intercalation, interfacial ion-transfer kinetics, and electrolyte-dependent chemistry [22,23,24].

The behavior in the acidic AlCl_3_ electrolyte provides additional support for this interpretation. As shown in Figure S5, although Al^3+^ has a higher charge density than Zn^2+^, the WO_3_·2H_2_O film still exhibits a pronounced electrochromic response under proton-rich conditions. Considering the acidic nature of the AlCl_3_ electrolyte, the response may involve proton-assisted Al^3+^-related charge compensation, or possible H^+^/Al^3+^ co-intercalation [25]. Thus, the AlCl_3_ result further supports the conclusion that pH regulation in hydrated WO_3_ is fundamentally cation-dependent, but cannot be reduced to cation valence alone.

This interpretation is also consistent with the kinetic analysis of the ZnCl_2_ system. The transition from mixed diffusion-controlled/capacitive behavior to more surface-confined kinetics can be understood in terms of the effective ion-accessible depth of the WO_3_·2H_2_O film. In the ZnCl_2_ electrolyte, increasing pH leads to *b* values approaching 1.0 and a decrease in *D*_app_, indicating that Zn^2+^-related charge compensation becomes increasingly limited to the surface or near-surface region. This interpretation is consistent with previous studies on nanostructured WO_3_ films, which showed that when ion transport into the film is limited, the electrochemical response becomes more surface-controlled rather than bulk-diffusion-involved [26]. Because the same electrodeposited films were used at different pH values, the observed kinetic transition is mainly attributed to electrolyte-controlled ion accessibility rather than changes in film morphology. Nevertheless, the granular nature of the electrodeposited film may affect the effective ion-transport length and the extent of bulk participation.

**Figure 7:**
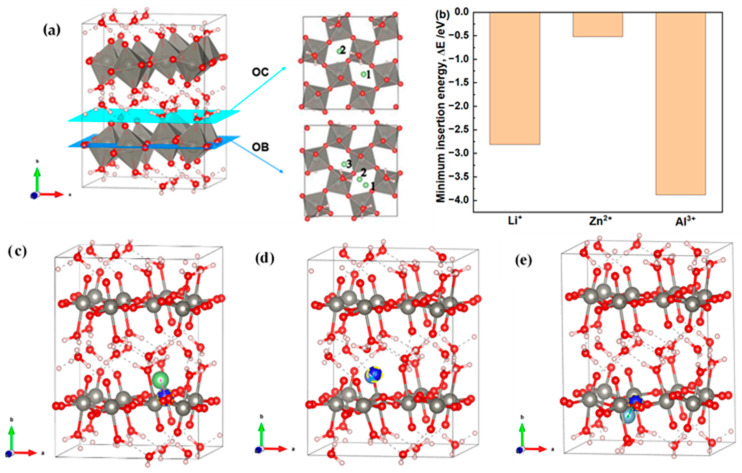
Structural model and cation–insertion configurations of WO_3_·2H_2_O used in the first-principles calculations. (**a**) Crystal structure of WO_3_·2H_2_O showing the intralayer framework and hydrated channels, together with the candidate insertion sites considered in the calculations. OB1–OB3 denote candidate sites within the intralayer framework, while OC1–OC2 denote candidate sites in the hydrated-channel region. (**b**) Minimum insertion energies of Li^+^, Zn^2+^, and Al^3+^ in WO_3_·2H_2_O after full structural relaxation. (**c**–**e**) Representative initial and relaxed positions of inserted Li^+^, Zn^2+^, and Al^3+^, respectively.

To further probe the intrinsic host-guest compatibility of different cations in the WO_3_·2H_2_O framework, first-principles calculations were performed by placing Li^+^, Zn^2+^, and Al^3+^ at different initial candidate sites. As shown in Figure 7a, the selected initial sites include intralayer framework sites and hydrated-channel sites. After structural relaxation, the inserted cations tend to stabilize in or near the interlayer/hydrated-channel regions (Figure 7c–e; Table S1). This result suggests that the hydrated framework provides favorable accommodation environments for inserted cations and may contribute to ion storage and structural adaptability.

The insertion energies calculated from the relaxed configurations are summarized in Figure 7b and Table S2. Li^+^ exhibits favorable insertion energetics, indicating good compatibility with the WO_3_·2H_2_O host. In contrast, Zn^2+^ shows weaker and more site-dependent insertion behavior, suggesting stronger sensitivity to the local coordination environment. This difference is consistent with the experimental observation that the ZnCl_2_ system is more strongly affected by pH-regulated ion accessibility and interfacial transfer kinetics than the LiCl system. Al^3+^ also shows favorable insertion energetics despite its higher charge, indicating that cation valence alone does not determine the cation–host compatibility or the electrochromic response.

Since solvation, interfacial desolvation, proton insertion, and proton-coupled charge compensation are not directly included, the DFT results serve only as a thermodynamic reference for intrinsic cation–host compatibility. The experimental differences among the Li^+^, Zn^2+^, and Al^3+^ systems should therefore be understood as the combined result of host-guest compatibility, ion accommodation within the hydrated framework, and pH-regulated ion accessibility.

Overall, these results indicate that the electrolyte pH regulates aqueous electrochromism in hydrated WO_3_ through coupled effects on surface protonation, proton-assisted charge compensation, interfacial ion-transfer kinetics, and cation-dependent electrolyte chemistry. The different responses of the Li^+^, Zn^2+^, and Al^3+^ systems show that the role of these factors depends strongly on the cation involved.

These findings also suggest possible strategies for overcoming the kinetic limitation observed at higher pH. Surface functionalization may help improve interfacial wettability, proton/cation transfer, and desolvation kinetics, while intentional nanoscale porosity or a more open nanostructure could shorten ion-diffusion pathways and increase accessible electroactive sites. However, excessive porosity may reduce optical transparency, weaken mechanical integrity, and accelerate cycling degradation. Therefore, future structural optimization should balance improved ion accessibility with optical and structural stability.

Although chloride-based electrolytes were used in this work to maintain a common anion background and clarify the effects of cation species and pH, their practical use in large-area electrochromic devices may raise concerns related to corrosion and electrolyte disposal. More environmentally benign sulfate- or acetate-based electrolytes could be considered in future studies. However, because changing the anion may also affect cation solvation, hydrolysis behavior, ionic conductivity, and interfacial charge-transfer kinetics [22], further systematic comparison is needed to balance electrochromic performance and environmental compatibility.

## 4. Conclusions

Amorphous WO_3_·2H_2_O films electrodeposited on ITO substrates exhibited pronounced pH-dependent electrochromic behavior in aqueous electrolytes, and this pH sensitivity was strongly dependent on the cation species. In the LiCl electrolyte, lower-pH conditions, especially pH 2.0, produced the largest optical modulation and the best cycling stability, while the charge-storage behavior remained largely pseudocapacitive throughout the investigated pH range. In the ZnCl_2_ electrolyte, lower-pH conditions within the acidic window significantly improved optical modulation, ion-transport kinetics, and cycling durability, with pH 2.0 providing the best overall balance among optical contrast, switching response, and charge utilization. Comparison of the Li^+^, Zn^2+^, and Al^3+^ systems indicates that electrolyte pH regulates not only proton-assisted charge compensation, but also the cation-dependent interfacial environment governing ion storage and transport in hydrated WO_3_. In particular, the influence of pH becomes more pronounced in multivalent-ion systems, where stronger hydration and greater transport limitations make the electrochromic response more sensitive to the electrolyte environment. Overall, this work demonstrates that pH control is an effective strategy for tuning the electrochromic performance of hydrated WO_3_ films in aqueous systems and provides useful guidance for the rational design of high-performance aqueous electrochromic electrolytes and devices.

## Figures and Tables

**Table 1:** Electrochemical and electrochromic performance of WO_3_·2H_2_O films in 1.0 mol/L LiCl electrolytes with different pH values.

pH	Δ*T* at 633 nm (%)	*t*_b_/*t*_c_ (s)	CE (cm^2^/C)	Areal Capacitance (mF/cm^2^)	Representative Cycling Retention ^1^
2.0	75.5	4.5/9.0	59.2	26.1	83.3% retention after 300 cycles
5.0	54.9	2.5/5.5	63.8	12.6	71.9% retention after 50 cycles
8.6	45.6	2.5/6.5	78.1	9.7	70.2% retention after 50 cycles

^1^ The retention values were measured after different representative cycle numbers because the degradation rates varied substantially with electrolyte pH.

**Table 2:** Electrochromic performance of WO_3_·2H_2_O films in 0.5 mol/L ZnCl_2_ aqueous electrolytes with different pH values.

pH	Δ*T* at 633 nm (%)	*t*_b_/*t*_c_ (s)	CE (cm^2^/C)	Areal Capacitance (mF/cm^2^)	Representative Cycling Retention ^1^
1.5	83.0	7.8/8.0	56.8	32.9	90.9% retention after 400 cycles
2.0	80.2	4.3/8.0	66.5	26.9	91.5% retention after 400 cycles
2.5	62.1	2.6/16.0	69.4	22.0	80.4% retention after 400 cycles
3.0	64.3	4.0/10.8	64.8	16.6	82.5% retention after 150 cycles

^1^ The retention values were measured after different representative cycle numbers because the degradation rates varied substantially with electrolyte pH.

## Data Availability

The original contributions presented in this study are included in the article/Supplementary Material. Further inquiries can be directed to the corresponding author.

## Supplementary Materials

The following supporting information can be downloaded at: https://www.mdpi.com/article/10.3390/ma19101943/s1. Figure S1: Representative XRD patterns of the bare ITO substrate and the as-deposited WO_3_·2H_2_O film on ITO. Figure S2: Cycling stability and post-cycling characterization of the WO_3_·2H_2_O film in 1.0 mol/L LiCl electrolytes at pH 5.0 and 8.6; Figure S3: Open-circuit stability of the WO_3_·2H_2_O film in 0.5 mol/L ZnCl_2_ electrolytes at representative pH values. Figure S4: Post-cycling electrochromic and morphological comparison of WO_3_·2H_2_O films in 0.5 mol/L ZnCl_2_ electrolytes at different pH values; Figure S5: Electrochemical and electrochromic performance of the WO_3_·2H_2_O film in 0.33 mol/L AlCl_3_ aqueous electrolyte (pH 2.3). Figure S6: Schematic illustration of the pH-dependent electric double layer structure at the WO_3_/electrolyte interface. Table S1: Relaxed positions of Li^+^ and Al^3+^ obtained from different candidate insertion sites in the WO_3_·2H_2_O supercell. Table S2: Insertion energies of Li, Zn, and Al at different candidate sites in the WO_3_·2H_2_O supercell.
