# Supplementary material for: pH-Regulated Cation-Dependent Electrochromism of Electrodeposited WO3·2H2O Films in Aqueous Electrolytes
Source: Materials (Basel). 2026 May 9;19(10):1943. doi: 10.3390/ma19101943 (PMC13208360; doi:10.3390/ma19101943)
Supplement: Supplementary file 1 [file materials-19-01943-s001.zip › materials-4293571-supplementary.pdf]

Supplementary Materials for

pH-Regulated Cation-Dependent Electrochromism of Electrodeposited $WO_3 \cdot 2H_2O$ Films in Aqueous Electrolytes

Ruoming Du, Aihua Yao*

School of Materials Science and Engineering, Tongji University, Shanghai 200092, China

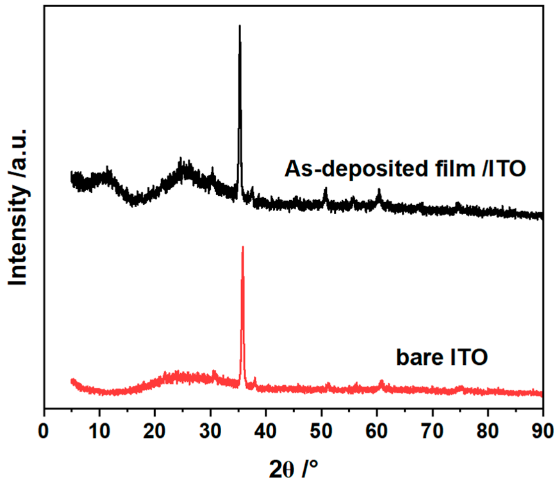

Figure S1. Representative XRD patterns of the bare ITO substrate and the as-deposited $WO_3 \cdot 2H_2O$ film on ITO

The XRD pattern shows broad low-angle features in addition to the reflections from the ITO substrate, with no distinct diffraction peaks attributable to crystalline WO_3. The broad feature centered at approximately 12.5° is consistent with the enlarged interlayer spacing associated with structural water in hydrated tungsten oxide. Therefore, the result supports the assignment of the as-deposited film as amorphous $WO_3 \cdot 2H_2O$, in agreement with our previous structural characterization based on the same electrodeposition protocol.

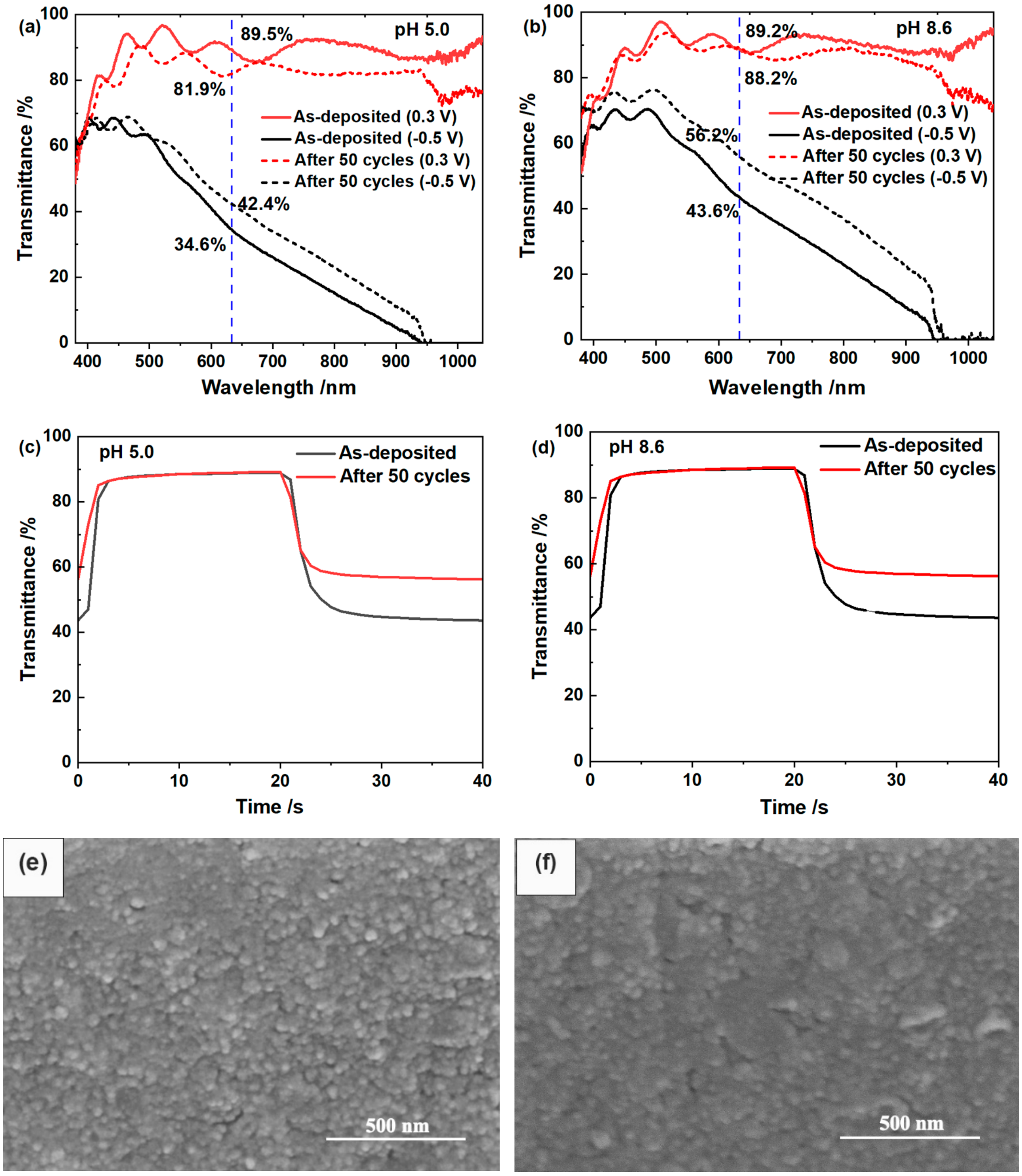

Figure S2. Cycling stability and post-cycling characterization of the $WO_3 \cdot 2H_2O$ film in 1.0 mol/L LiCl electrolyte at pH 5.0 and 8.6. (a,b) In situ transmittance spectra of the as-deposited film and the film after 50 cycles in the bleached (+0.3 V) and colored (-0.5 V) states. (c,d) Real-time transmittance response at 633 nm before and after 50 cycles. (e,f) SEM images of the films after 50 cycles.

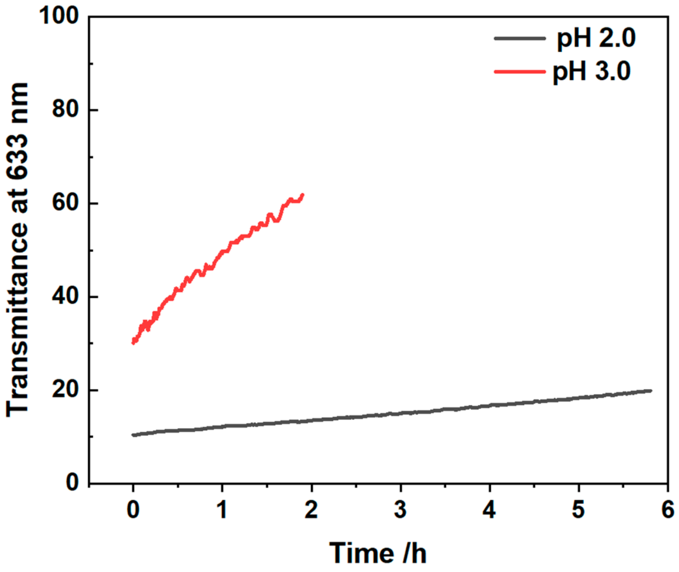

Figure S3. Open-circuit stability of the $WO_3 \cdot 2H_2O$ film in 0.5 mol/L $ZnCl_2$ electrolytes at representative pH values. The films were first colored at −0.5 V, after which the external bias was removed and the transmittance at 633 nm was monitored as a function of time under open-circuit conditions.

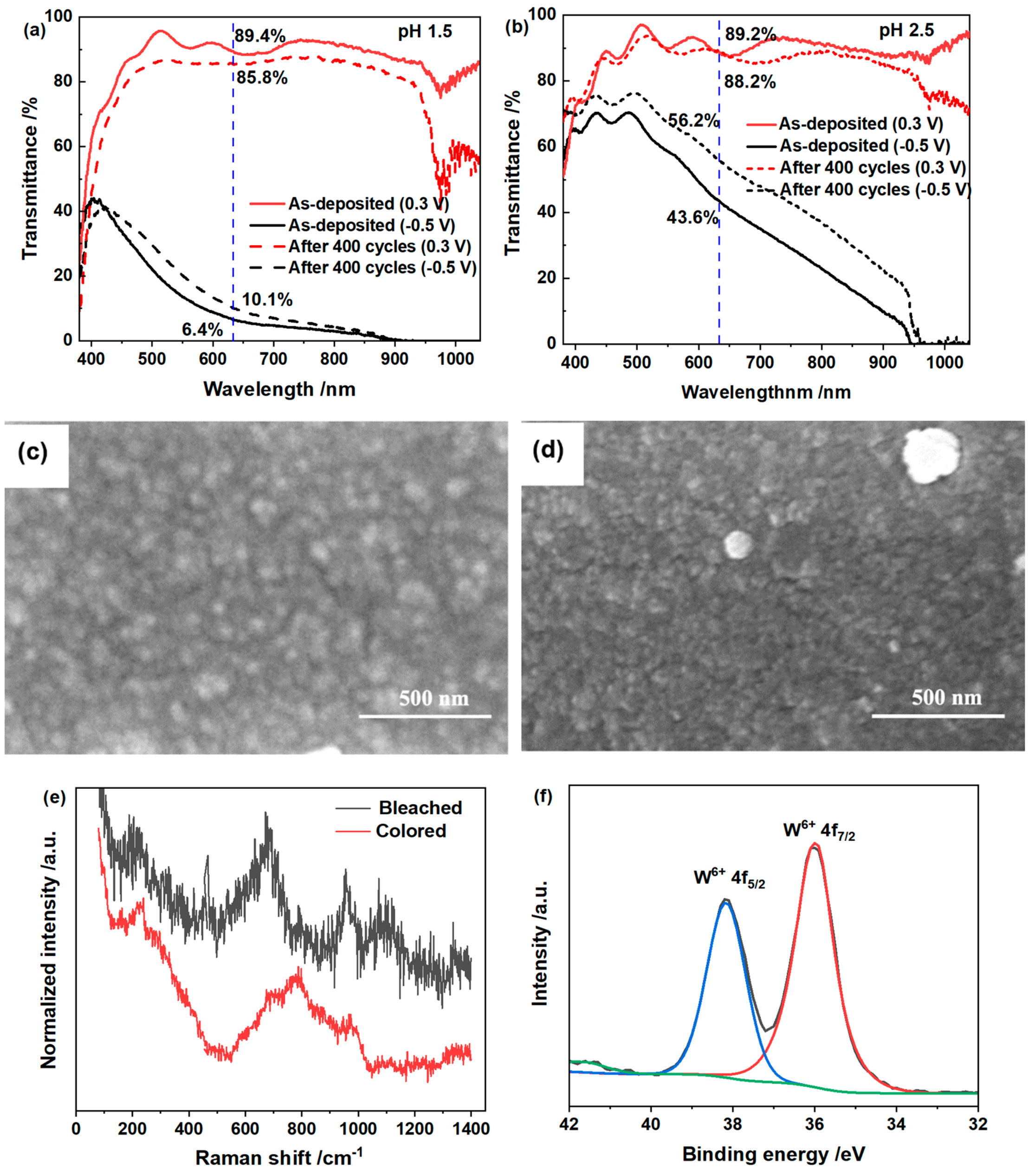

Figure S4. Post-cycling electrochromic and morphological comparison of $WO_3 \cdot 2H_2O$ films in 0.5 mol/L $ZnCl_2$ electrolytes at different pH values. (a) In situ transmittance spectra of the as-deposited film and the film after 400 cycles in the bleached (+0.3 V) and colored (-0.5 V) states at pH 1.5. (b) In situ transmittance spectra of the as-deposited film and the film after 400 cycles in the bleached (+0.3 V) and colored (-0.5 V) states at pH 2.5. (c, d) SEM images of the cycled films at pH 1.5 and 2.5, respectively. (e) Normalized Raman spectra and (f) W4f XPS spectrum of the film after 400 cycles at pH 1.5.

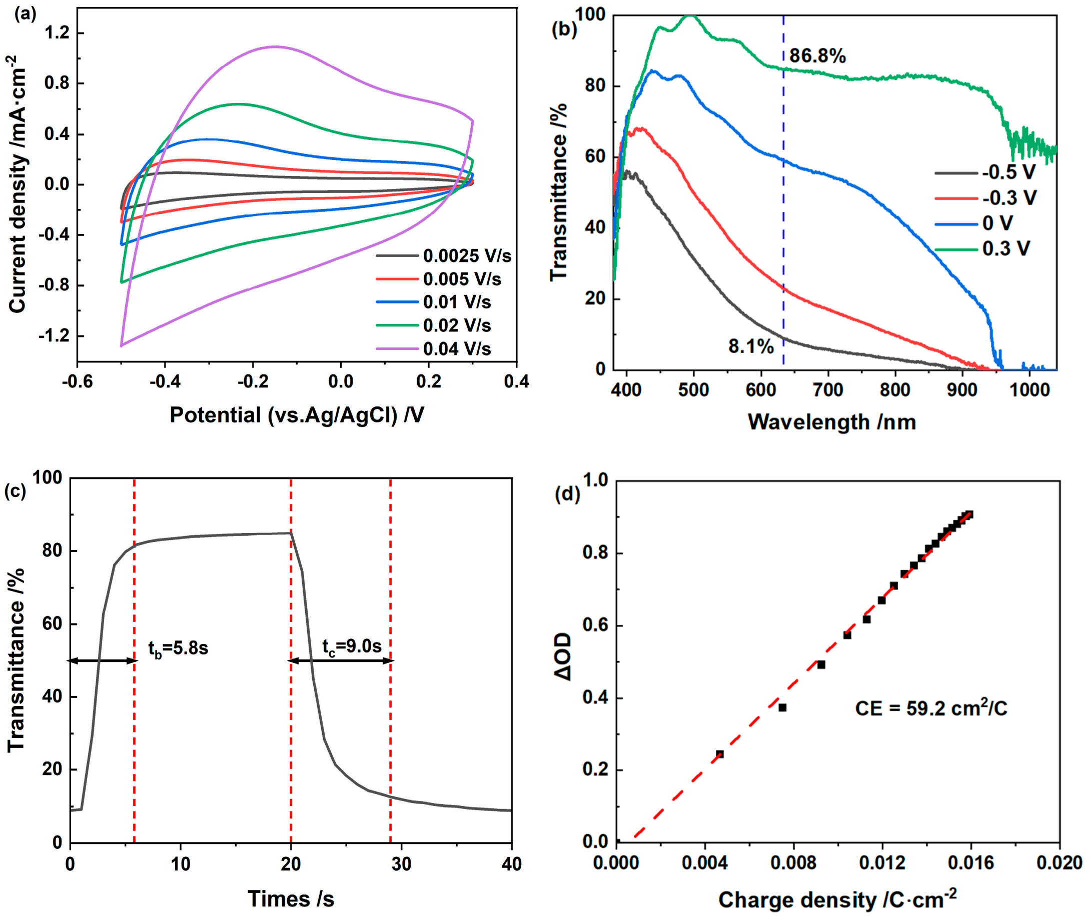

Figure S5. Electrochemical and electrochromic performance of the $WO_3 \cdot 2H_2O$ film in 0.33 mol/L $AlCl_3$ aqueous electrolyte (pH 2.3). (a) CV curves at different scan rates. (b) In situ transmittance spectra collected under different applied potentials. (c) Real-time transmittance response at 633 nm. (d) Coloration efficiency plot.

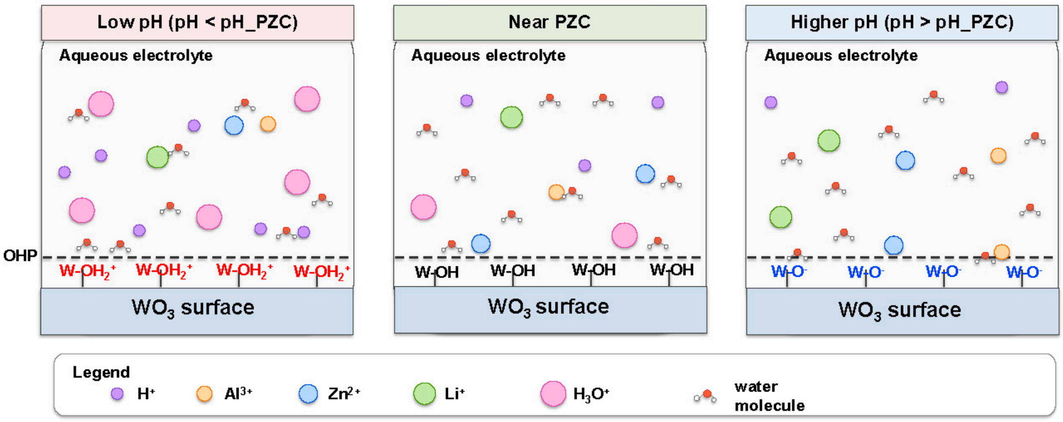

Figure S6. Schematic illustration of the pH-dependent electric double layer structure at the WO_3/electrolyte interface. At low pH, the surface is protonated and represented by W-OH_2^+ groups. Near the PZC, the surface is mainly neutral (W-OH). At higher pH, the surface becomes deprotonated (W-O^-), which alters the interfacial ion distribution and the cation desolvation environment.

First-principles calculations

Structural Model of $WO_3 \cdot 2H_2O$

Because a fully resolved experimental crystal structure for $WO_3 \cdot 2H_2O$ is not readily available in standard crystallographic databases, the initial atomic model was constructed on the basis of the isostructural $MoO_3 \cdot 2H_2O$ framework reported in the Crystallography Open Database (COD entry 9011122). This structural model has been adopted in previous first-principles studies of hydrated tungsten oxide and provides a reasonable starting point for describing the layered hydrated framework.

After structural relaxation, the optimized $WO_3 \cdot 2H_2O$ model exhibits hydrated interlayer galleries confined by terminal oxygen atoms of the WO_6 octahedral layers. In the present work, this hydrated interlayer region was regarded as the key structural environment for evaluating the insertion behavior of different cations.

Computational Details

First-principles calculations were performed to evaluate the intrinsic insertion behavior of Li, Zn, and Al in hydrated $WO_3 \cdot 2H_2O$. A fully relaxed $(WO_3 \cdot 2H_2O)_8$ supercell was used as the host model. Five symmetry-inequivalent candidate insertion sites were considered, including three sites in the intralayer framework (OB1, OB2, and OB3) and two sites in the hydrated-channel region (OC1 and OC2).

For each in species (M = Li, Zn, Al), a single ion was placed at each candidate site, and full structural relaxation was performed to identify locally stable configurations. The ion insertion energy was calculated according to:

$$\Delta E = E[M(WO_3 \cdot 2H_2O)_8] - E[(WO_3 \cdot 2H_2O)_8] - \mu_M$$

where $E[M(WO_3 \cdot 2H_2O)_8]$ and $E[(WO_3 \cdot 2H_2O)_8]$ are the total energies of the ion-inserted and pristine supercells, respectively, and μ_M is the chemical potential of the inserted species. The chemical potentials were referenced to the corresponding elemental bulk phases. A negative ΔE indicates thermodynamically favorable insertion, whereas a less negative or positive value indicates weaker or unfavorable insertion tendencies.

All calculations were performed within the framework of density functional theory using the Vienna *Ab initio* Simulation Package (VASP). The electron–ion interaction was described using the projector augmented-wave (PAW) method, and the exchange–correlation energy was treated using the Perdew–Burke–Ernzerhof (PBE) functional. The plane-wave cutoff energy was set to 650 eV, and Brillouin-zone integration was carried out using a 3× 3 × 3 Monkhorst–Pack *k*-point mesh. Structural optimization was continued until the total-energy change was below 1×10^{-5} eV and the residual atomic forces on all atoms were below 0.02 eV/Å.

The calculated insertion energies at all examined candidate sites are summarized in Table S1. Li^+ and Al^{3+} exhibit favorable insertion energies at multiple sites, whereas Zn^{2+} shows weaker and more

site-dependent insertion behavior. The fully relaxed positions of Li+ and Al3+ initially placed at different candidate sites are listed in Table S2.

Table S1. Relaxed positions of Li^+ and Al^{3+} obtained from different candidate insertion sites in the $WO_3 \cdot 2H_2O$ supercell

Ion	Site	Relaxed position (x, y, z)
Li^+	OB1	(0.628, 0.352, 0.375)
Li^+	OB2	(0.628, 0.352, 0.374)
Li^+	OB3	(0.370, 0.288, 0.613)
Li^+	OC1	(0.365, 0.396, 0.588)
Li^+	OC2	(0.630, 0.356, 0.381)
Al^{3+}	OB1	(0.629, 0.331, 0.365)
Al^{3+}	OB2	(0.628, 0.332, 0.365)
Al^{3+}	OB3	(0.372, 0.168, 0.614)
Al^{3+}	OC1	(0.383, 0.312, 0.565)
Al^{3+}	OC2	(0.628, 0.332, 0.365)

Several candidate insertion sites converge to closely related interlayer or near-interlayer positions after structural relaxation.

Table S2. Insertion energies of Li, Zn, and Al at different candidate insertion sites in the $WO_3 \cdot 2H_2O$ supercell

Ion	Site	Region	Insertion energy, ΔE (eV)	Relative stability
Li^+	OB1	Intralayer framework	-2.814	Most favorable
Li^+	OB2	Intralayer framework	-2.810	
Li^+	OB3	Intralayer framework	-2.237	
Li^+	OC1	Hydrated channel	-2.694	
Li^+	OC2	Hydrated channel	-2.801	
Zn^{2+}	OB2	Intralayer framework	-0.394	
Zn^{2+}	OB3	Intralayer framework	-0.390	
Zn^{2+}	OC1	Hydrated channel	-0.520	Most favorable
Zn^{2+}	OC2	Hydrated channel	4.485	Least favorable
Al^{3+}	OB1	Intralayer framework	-3.862	
Al^{3+}	OB2	Intralayer framework	-3.871	
Al^{3+}	OB3	Intralayer framework	-3.881	Most favorable
Al^{3+}	OC1	Hydrated channel	-2.760	
Al^{3+}	OC2	Hydrated channel	-3.864	

For Zn^{2+}, the OB1 site was also examined as an initial candidate insertion site. However, this configuration was found to be unstable during structural relaxation, and no meaningful converged insertion-energy value could therefore be obtained. For this reason, the OB1 site is not listed in Table S1.
